# Temporary Portocaval Shunts During Liver Transplantation: A Narrative Review of Technical Solutions and Post-Transplant Outcomes

**DOI:** 10.3390/jcm14248723

**Published:** 2025-12-09

**Authors:** Elisa Schirra, Alberto Mauro, Giuseppe Bianco, Marco Maria Pascale, Francesco Frongillo, Erida Nure, Alfonso Wolfango Avolio, Salvatore Agnes, Gabriele Spoletini

**Affiliations:** 1 General Surgery and Transplantation Unit, Fondazione Policlinico Universitario Agostino Gemelli IRCCS, 00168 Rome, Italy; 2 Faculty of Medicine and Surgery, Università Cattolica del Sacro Cuore, 00168 Rome, Italy

**Keywords:** liver transplant, portocaval shunt, portal flow preservation, hemodynamic stability

## Abstract

**Background**: Liver transplantation (LT) continues to evolve with techniques aimed at minimizing perioperative complications associated with caval and portal vein clamping. Caval-sparing approaches, such as the piggyback technique, preserve hemodynamic stability; however, portal clamping remains necessary and may trigger postreperfusion syndrome, endotoxemia, and hepatic microcirculatory disturbances. Temporary portocaval shunts (PCSs) have been developed to maintain portal flow during LT, mitigating these adverse effects and allowing for hemodynamic stability and a reduced intraoperative bleeding. **Portocaval Shunts**: Various PCS techniques—including end-to-side, right-branch, portosaphenous, mesenterico-saphenous, iliac-venous conduit interposition, portoumbilical, and Rex-saphenous shunts—allow an individualized approach based on patient anatomy and surgical complexity. **Review of Evidence**: Available evidence demonstrates that PCS improves intraoperative hemodynamic stability, reduces blood transfusion requirements, and preserves renal function, particularly in patients with high portal flow or severe portal hypertension. PCS may also shorten warm ischemia time, facilitate hepatectomy, and enhance outcomes in extended criteria donor grafts or marginal organs. Meta-analyses and randomized studies support its role in reducing intraoperative blood loss, improving early graft function, and accelerating postoperative recovery. However, the effect of PCS on long-term survival and major postoperative morbidity remains variable, likely due to heterogeneity in patient populations, donor types, and perioperative management. **Conclusions**: Overall, PCS represents a safe and feasible adjunct in LT, offering significant hemodynamic and technical advantages. Its use should be individualized based on patient risk factors, intraoperative hemodynamics, and anticipated intraoperative challenges. PCS provides a practical strategy to preserve portal flow, minimizing intraoperative complications and facilitating the hepatectomy. However, the decision to create a PCS during LT still depends on the surgeon’s preference. Postoperative outcomes and impact on long-term survival require further investigation.

## 1. Introduction and Background

Liver transplantation (LT) is a constantly evolving surgical procedure. Historically, the first liver transplants entailed a veno-venous bypass (VVB) due to the necessity of clamping the infrahepatic and suprahepatic vena cava, as recipient hepatectomy involved the removal of the retrohepatic cava and fashioning two end-to-end anastomoses for caval reconstruction [1]. To avoid the subsequent hemodynamic instability and the requirement of a VVB (along with prolongation of the surgical time and the risk of thromboembolic complications [2]), caval-sparing techniques were introduced. The best known and most commonly adopted is the piggyback technique [3]. Subsequently modified by Belghiti [4], it consists of an anastomosis between a cuff of the graft hepatic veins and the recipient vena cava, which can be performed without obstructing caval flow, and applying only partial caval clamping. 

While these techniques allow for the preservation of the recipient vena cava, with a consequent benefit on hemodynamic stability, portal clamping is still required.

Portal clamping also has its own downsides, namely, blocking splanchnic venous outflow and increasing the risk of postreperfusion syndrome, endotoxemia, and bacterial translocation during LT. 

In a study published by Marzi et al. on animal models [5], when the portal vein (PV) was clamped during LT in rats, the intestines received a reduced blood supply, releasing harmful inflammatory signals. These signals led to the activation of the immune system, causing increased leukocyte adhesion in liver sinusoids and disturbance of hepatic microcirculation in the transplanted liver. Interestingly, when a portosystemic shunt was used, it mitigated these adverse effects, suggesting that endotoxin release from the gut and activation of hepatic macrophages may drive chemoattractant mediator release and leukocyte–endothelial activation. While these data derive from animal models and cannot be translated directly to humans, they highlight a potential factor to consider in liver transplantation, especially with marginal grafts which are intrinsically more susceptible to ischemia–reperfusion injury, which could be exacerbated by endotoxemia.

Paugam-Burtz et al., in their prospective study [6], identified portal clamping as an independent risk factor of developing postreperfusion syndrome (PRS) during LT. They defined PRS as a drop in mean arterial pressure of >30% compared with the anhepatic stage, lasting more than 1 min, during the first 5 min after graft reperfusion. In their multivariable analysis, the absence of a PCS during surgery was found to be a predictor of PRS. Those patients were more prone to postoperative renal failure and had a lower early survival rate.

To mitigate these intra- and postoperative complications, a temporary PCS was introduced to preserve portal flow during LT: firstly described by Tzakis et al. [7] and then by Belghiti et al. [8], it gradually became widely accepted.

Our study aims to provide a review of the procedural aspects involving PCS and its effects on major post-LT outcomes and an overview of alternative and less common PCS techniques which so far remain underrepresented and inconsistently reported. This narrative review, despite the heterogeneity in available data and the paucity of prospective studies, provides a useful outline of both clinical and technical applications for everyday surgical practice.

Veno-venous bypass, which also redirects splanchnic flow to the systemic circulation, differently from passive flow PCS techniques, requires a pump-driven extracorporeal circulation and will not be the object of our discussion.

## 2. Portocaval Shunts

### 2.1. Direct PCS

A ***PCS*** is generally created after full or partial dissection and isolation of the PV and the inferior vena cava (IVC). The PV is then divided at the portal bifurcation, and a longitudinal venotomy is made on the anterior wall of the infrahepatic IVC. Both openings should be of similar diameter to avoid turbulence or stenosis. An end-to-side anastomosis is performed, typically using a continuous running suture with 4-0 or 5-0 polypropylene [9]. 

A possible technical variation is a ***right PCS*** [10], performed by fashioning an anastomosis between the right branch of the PV and the anterior surface of the IVC: this technique could prevent excessive shortening of the PV, avoiding extensive dissection and large openings of the IVC thanks to the smaller caliber of the right PV branch. This solution may be easier due to the axis and the proximity of the right portal branch to the IVC, though it still requires dissection of both portal branches and ligation of the left branch. 

Another described variant of the portocaval anastomosis, which is quick and theoretically simple, involves positioning a plastic ***cannula*** in the PV free-end, after transection, and connecting it to the IVC after a small venotomy [11]. 

Moreover, an ***iliac-venous conduit interposition*** has been proposed as another possible option for patients with a challenging portocaval anastomosis [12]: it is performed retrieving an iliac graft from the donor, which is then anastomosed to both the IVC and the PV; this graft may be preserved to lengthen a short native PV.

Ultimately, the choice among these variations depends on the surgeon’s preference and experience, as no approach has been proven superior to the other.

Once graft implantation begins, after creating the cavo-caval anastomosis, the shunt is quickly interrupted (generally dividing it with a mechanical stapler), and the recipient’s PV is ready for the end-to-end anastomosis.

### 2.2. Ex Situ Portal-Venous Shunts

A portocaval anastomosis is not the sole option to maintain portal flow during transplant. Passive (i.e., without the need for an external pump) ex situ portal-venous shunts have been described too. 

These indirect ex situ PCS, although less common, could prove useful when operating on patients with a technically difficult dissection of the hepatic pedicle, such as in the case of a large segment I, portal thrombosis or cavernomatosis, re-transplantation, or previous supramesocolic surgery, in which a direct PCS could be more demanding [13,14].

Pratschke et al. described their routinary use of an extracorporeal shunt catheter connecting the PV and the ***femoral vein***, to establish a spontaneous temporary blood flow [15]: this procedure is performed by cannulating the femoral vein by direct puncture (17 Fr) and the portal vein free end by insertion of a 24 Fr cannula which is then secured by tourniquet ligation; the portosystemic pressure difference then allows for spontaneous circulation. Addeo et al. use a similar technique, the ***portosaphenous shunt***, in the case of patients who could benefit from an indirect shunt and who have a difficult access to the inferior mesenteric vein or to the hepatic pedicle [14]: this technique similarly entails cannulation of the saphenous vein with a 18F cannula, which is then connected to the cannula that drains the PV.

In the case of a challenging dissection of the hepatic pedicle, which could render the PV approach time-consuming and dangerous, a possible option is a ***mesenterico-saphenous shunt***, which is performed by cannulating and connecting both the inferior mesenteric vein and the saphenous vein. This technique was documented to obtain similar hemodynamic stability and efficiency in decompressing the splanchnic circulation and positive short-term outcomes compared to standard PCS, with a much faster learning curve [13], and some centers use it routinely in case of foreseen difficulties in pedicle dissection, such as in re-LT, hypertrophic segment I, obese patients [14], previous supramesocolic surgery, portal thrombosis, or portal cavernomatosis [13].

**Figure 1:**
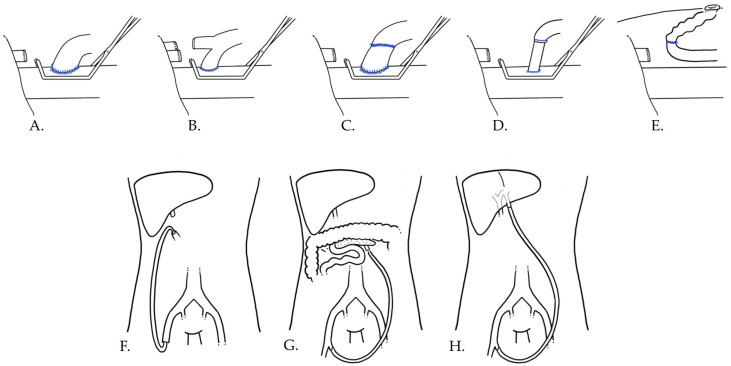
Schematic representation of direct and ex situ PCS. (**A**) Portocaval anastomosis. (**B**) Right portocaval anastomosis. (**C**) Iliac-venous conduit interposition. (**D**) Shunt to IVC via portal cannulation. (**E**) Portoumbilical anastomosis. (**F**) Porto-femoral shunt. (**G**) Meso-saphenous shunt (via IMV cannulation). (**H**) Rex-saphenous shunt.

Lastly, some patients with cirrhosis and severe portal hypertension show ***recanalization of the umbilical vein***: the simple preservation of this vein and its collaterals, which are generally ligated at the beginning of the hepatectomy, could represent a useful method to maintain a spontaneous shunt and successfully reduce portal pressure during the hepatectomy phase [16]; similarly, it is possible to exploit a large recanalized umbilical vein to perform a ***portoumbilical anastomosis***, which has been described as a possible efficient method for achieving a quick portosystemic shunt [17]. Another option is the ***Rex-saphenous shunt***, connecting the umbilical and saphenous vein via cannulas [18]. Notably, the latter option has been deemed to be useful only in the case of proven hepatofugal flow on preoperative Doppler ultrasound, suggesting that ligating the umbilical vein could have an effect comparable to PV clamping. The recanalized umbilical vein, originating from the Rex recess, connects the portal system to the caval circulation via the external iliac veins [18]. As the last step during hepatectomy, the cannula in the umbilical vein is moved to the sectioned PV, converting the Rex-saphenous shunt into a portosaphenous shunt [18]. These techniques are summarized in Figure 1 and Table 1.

## 3. Clinical Impact of PCS During LT: A Critical Review of Evidence

Theoretically, patients who could benefit most from preserving portal flow are those with no collateral circulation, such as patients with fulminant hepatitis [19], rather than patients with cirrhosis who commonly have a plurality of portosystemic collaterals which could take over after portal clamping.

Nonetheless, its impact has been widely examined also in the setting of cirrhotic patients undergoing LT. The main outcomes analyzed could be grouped together as follows:

- Hemodynamic stability and preserved renal function;
- Duration of the entire surgical procedure;
- Blood transfusion requirement;
- Survival rate.

We analyzed the available literature, mainly consisting of retrospective studies with varied populations and criteria, which offers an overview of the potential benefits and indications of PCS.

Figueras et al. [20] performed a prospective randomized study which documented a significant positive effect of PCS on maintaining ***hemodynamic*** ***stability*** and preserving ***renal function*** during the anhepatic stage; in particular, they found an even greater effect with shunts performed on patients with severe portal hypertension (specifically patients with a baseline portal flow of 1000 mL/min or greater and/or a portocaval gradient of 16 mm Hg or greater), registering fewer blood transfusions, better hemodynamics, and more stable urinary output compared to those without PCS. Moreover, patients in the PCS group had a shorter artificial ventilation time and shorter ICU stay. 

Other series, such as the single-center analysis by Ghinolfi et al. [21], confirmed the association of PCS with a better hemodynamic stability and a transiently improved creatinine clearance, as well as an early (30-day) survival of both graft and patient, which, however, was lost at 3 months. 

The retrospective study by Margarit et al. [22], also focusing on cirrhotic patients, highlighted a positive effect of PCS (reduced ***blood transfusion requirements*** and better postoperative renal function) in patients with an initial portal vein flow > 800 mL/min, while patients with a PV flow < 800 mL/min did not show any significant benefit from PCS. 

Nonetheless, not all studies on the topic agree with these findings: Pietersen et al. [23] found no significant reduction in the onset or in the severity of acute kidney injury following LT.

***The duration of the entire surgical procedure*** is usually not impacted by the creation of a temporary PCS; on the contrary, a shorter surgical duration has been reported, as time spent on the portocaval anastomosis is regained later on during the hepatectomy, which is simpler and faster thanks to the liver devascularization which eases the hepatocaval dissection and favors a bloodless surgical field [20,21,24,25]. However, while PCS does not significantly prolong surgical time [24], it was reported to shorten warm and total ischemia time [26]: this advantage could be even more relevant in the context of extended-criteria donors (ECDs).

Grafts from ***ECDs***, which are currently routinely accepted in an effort to widen the donor pool, are more prone to ischemia–reperfusion injury (IRI), carrying a higher risk of peri- and postoperative complications, including early allograft dysfunction (EAD) [27]. In patients receiving an ECD graft, performing a temporary PCS has been shown to improve graft survival and early graft recovery (i.e., reduced biliary complications, improved postoperative liver function, and reduced blood transfusion requirement) [28]. Indeed, ECD grafts could be more susceptible to the hemodynamic instability and noxious agents developed during portal clamping [21]. 

Notably, Pratschke et al. [15] observed an improved long-term graft survival (with a documented hazard ratio of 2.1), especially for patients with high MELD scores and for those receiving a marginal organ, as well as a decreased re-transplantation rate. 

A favorable ***survival rate*** using PCS has also been demonstrated for patients receiving a “standard” graft. PCS showed a protective effect, reducing first-year mortality by 70% (in a cohort of selected patients with no chronic kidney disease) [29]. Moreover, better overall survival was reported in a retrospective series by Nacif et al. [24].

Another possible advantage of PCS is its use in cases of portal thrombosis: after thrombectomy, PCS could maintain an appropriate portal blood flow to possibly avoid rethrombosis and evaluate the efficacy of the thrombectomy itself [22]. However, to our knowledge, this possibility has not yet been evaluated directly in any retrospective or prospective data analysis.

### Evidence from Meta-Analyses and Randomized Clinical Trials

Several meta-analyses have been published to clarify the benefits of PCS during and after LT. Pratschke et al. [30], in their analysis of six studies, described a statistically significant effect on blood transfusion requirements and on creatinine levels on the 3rd postoperative day. The reduced requirement for blood transfusions could be interpreted as a reduced intraoperative blood loss, translating as a positive effect on both the surgical complexity and the patient’s condition. The results on kidney function are not easily interpreted, due to several variables that could be confounders (i.e., preoperative degree of portal hypertension and preoperative kidney function). This analysis also found a statistically significant effect on the reduction in AST levels in patients receiving a PCS, which can be interpreted as an indicator for postreperfusion syndrome. On the other hand, no statistically significant effect was found on primary non-function, ALT values, length of surgery, requirement of fresh frozen plasma, and length of hospital stay.

A more recent meta-analysis from Nacif et al., published in 2018 [24], emphasized a statistically significant effect of temporary PCS on length of hospital stay, incidence of primary non-function, and mortality; however, no significant difference was found regarding length of surgery, ALT levels, re-transplant, third-postoperative-day serum creatinine, blood, fresh frozen plasma, or platelet transfusion requirements.

It should be underlined that the authors also included three studies investigating the role of hemi-PCS, which is more commonly performed in living donor transplants, and therefore results should be considered with caution for the purpose of this article.

The most recent work available is a meta-analysis from Mataruco et al., published in August 2025 [31], comprising 13 studies (2 RCTs and 11 observational studies): according to their results, temporary PCS has a statistically significant effect on both intraoperative parameters (the amount of red blood cell units transfused) and postoperative morbidity (creatinine levels 72 h after LT, biliary complications, and Clavien–Dindo complication score ≥ 3). On the contrary, graft and patient survival (despite a longer anhepatic phase in the PCS group), blood loss, platelet and fresh frozen plasma use, red blood cell transfusion requirement, need for renal replacement therapy, length of surgery, and ICU and hospital stay were not affected using the temporary shunt. 

It is interesting to note how the use of PCS only affects the amount of red blood cell units, while the absolute need for blood products is not impacted.

These findings seem to suggest that PCS should be taken into consideration especially for patients at higher risk of biliary complications or intraoperative bleeding. 

Lastly, it should be mentioned that a randomized clinical trial by Yl et al. in 2024 [32] reported a significant advantage for patients receiving a PCS: considering intraoperative parameters, patients required less hemodynamic support during the anhepatic phase (and thus had improved hemodynamic stability) and fewer red blood cell, platelet, and plasma transfusions (along with reduced intraoperative bleeding); they also had fewer IVC injuries, a higher urine output, and a shorter duration of surgery (despite a longer anhepatic phase, with comparable ischemia time) compared to the no-PCS cohort. With respect to postoperative results, patients who received a PCS experienced a faster serum lactate normalization (after reaching a comparable peak level at 24 h post-surgery) and were able to tolerate enteral feeding on average a day earlier. Conversely, overall major morbidity, incidence of acute kidney injury, serum bilirubin normalization time, and development of ascites were comparable between the PCS and no-PCS cohort. These results highlight the effect of PCS on intraoperative variables, which are reportedly more influenced; as far as postoperative outcomes are concerned, there are likely more factors at play that hamper a possible positive effect of PCS.

We summarize the main outcomes from the aforementioned studies in Table 2.

## 4. Discussion

While temporary PCS clearly improves intraoperative hemodynamics and some early postoperative parameters, such as faster lactate normalization and earlier tolerance of enteral feeding, its impact on major postoperative morbidity, acute kidney injury, and long-term survival is less consistent. In fact, available studies are mostly retrospective and heterogeneous with respect to population size and characteristics, study design, surgical technique, and endpoint definition, which hampers drawing clear results in terms of temporary PCS benefit. Nonetheless, despite limitations, the use of PCS is backed by its immediate benefit during hepatectomy in terms of hemodynamic stability and reduced intraoperative bleeding, with an acceptable cost in terms of surgical time and with little-to-no risk for the patient, as available data report neither morbidity nor mortality linked to the use of temporary PCS.

Indeed, temporary PCS represents a valuable adjunct in LT, particularly for patients at high risk of portal hypertension, marginal grafts, or significant intraoperative bleeding. Its main advantages include enhanced hemodynamic stability, reduced intraoperative bleeding, improved early renal and graft function, and facilitation of hepatectomy, while its effect on long-term outcomes remains limited. PCS is one component among many that influence the complex perioperative course of liver transplantation; it facilitates a bloodless hepatectomy without prolonging surgical time, providing a technical advantage, especially for less experienced surgeons, by reducing the risk of bleeding and IVC injury.

As far as our center experience is concerned, we routinely perform a classical end-to-side portocaval anastomosis for patients with severe portal hypertension causing large collaterals and ascites, in the case of a foreseeably difficult hepatectomy, to limit intraoperative blood loss, and in the case of fulminant hepatitis (in which patients typically lack collateral circulation). In our practice, the main scope of performing a PCS is to improve intraoperative outcomes; we found such a procedure to be helpful and simple to perform, granting us a technical advantage for a safer hepatectomy. For cases deemed worthy of fashioning a PCS, we employ the standard technique performing a temporary portocaval anastomosis. So far, we have not encountered cases requiring PCS in which such a technique was not feasible. Of note, we sporadically use VVB for cases in which caval preservation is not feasible.

We summarize the main indications for PCS in Table 3.

Desirably, large cohort prospective studies could fill the gap in defining which patients would benefit the most from PCS, as available studies are often contradictory and do not harbor the required statistical power to define indications for and benefits of the procedure, particularly regarding postoperative outcomes. Nevertheless, PCS remains a feasible, safe, and harmless option to consider during liver transplantation. Ultimately, the decision to create a PCS should be left to the surgeon’s judgment, taking into account patient characteristics, intraoperative risks, and anticipated technical challenges during the hepatectomy.

## Figures and Tables

**Table 1:** Categorization of direct and ex situ PCS.

Direct PCS	Ex Situ PCS
Portocaval end-to-side anastomosis Right portocaval end-to-side anastomosis Shunt to the IVC via portal cannulation Shunt via iliac-venous conduit interposition	Shunt to the femoral or saphenous vein via portal cannulation Mesenterico-saphenous shunt (on cannula) Portoumbilical anastomosis Rex-saphenous shunt (on cannula)

**Table 2:** Main outcomes of the analyzed studies.

Study	Study Type	Patient Characteristics	Outcomes: PCS vs. No PCS		
			Positive	Neutral	Negative
Figueras et al., 2001 [20]	Prospective randomized, single-center	Cirrhotic patients, excluding re-LT, IVC resection, PV thrombosis, hepatorenal syndrome, pulmonary hypertension, and previous portosystemic shunts.	Cardiac output during anhepatic phase (particularly in patients with severe portal hypertension); Absolute need for RBC transfusions; Urinary output in the anhepatic phase (particularly in patients with severe portal hypertension); ICU stay; Ventilatory support duration; Serum creatinine in the first 72 h.	Surgical time; Reperfusion syndrome; Amount of RBC transfusions; Urinary output during LT; Hospital stay; Liver biochemical parameters in the first 72 h.	Anhepatic phase duration.
Margarit et al., 2005 [22]	Retrospective, single-center	Cirrhotic patients, excluding re-LT and prior portosystemic shunts.	RBC transfusion requirements and postoperative renal function, only in patients with high portal venous flow (>800 mL/min).	Surgical time (LT and anhepatic phase duration).	–
Arzu et al., 2008 [26]	Retrospective, single-center	Any indication for LT, excluding re-LT, patients receiving a split liver, “classic” piggyback technique *.	Warm and total ischemia time; Serum creatinine at discharge; Systolic arterial pressure at reperfusion, diastolic arterial pressure in the anhepatic phase, and reperfusion; Overall survival.	Amount of RBC and FFP transfusions; Blood loss; Urinary output; Hospital stay; Surgical time; Central venous pressure and pulmonary artery pressure in the anhepatic phase and at reperfusion; Serum creatinine at 24 h and 72 h.	–
Ghinolfi et al., 2011 [21]	Retrospective, single-center	Any indication for LT, excluding re-LT and patients with prior portosystemic shunts.	RBC transfusion requirement; Phenylephrine requirement; Serum creatinine in the first 72 h; 30-day mortality; Graft loss at one and three months, stratified for high DRI and high D-MELD.	Surgical time; Absolute need for RBC transfusions and amount of FFP and platelet transfusion requirements; Epinephrine and norepinephrine requirements; Length of hospital stay; Post-op liver function tests; 90-day mortality; Graft loss stratified for MELD.	–
Pratschke et al., 2013 [15]	Retrospective, single-center	Any indication for LT, excluding patients receiving a split liver.	Vasopressor requirements; AST/ALT levels up to 7th POD; Early graft loss and re-LT; Long-term graft survival (especially for MELD ≥ 35 and DRI ≥ 1.25).	Amount of RBC and FFP transfusions; Serum creatinine levels up to 7th POD.	–
Rayar et al., 2017 [28]	Retrospective, single center	Any indication for LT, excluding re-LT, ALF and patients who could not receive a PCS due to preexisting conditions, with prior portosystemic shunt, living donors, or split liver.	Absolute need and amount of RBC and FFP transfusions; Post-op GGT, alkaline phosphatase, INR, and PT values; Incidence of biliary complications; 3-month graft survival rate (particularly for ECD grafts); Overall graft survival (only in subgroup analysis, for donors over 70 y).	Cold ischemia time; Absolute need and amount of platelet transfusions; Post-op AST/ALT and bilirubin levels; Incidence of arterial complications; Post-op renal function; Incidence of Clavien–Dindo complication score ≥3; ICU and hospital stay; 3-month graft survival rate for non-ECD grafts; Overall graft survival.	Surgical time; Anhepatic phase duration.
Tortolero et al., 2020 [29]	Retrospective, single-center	Any indication for LT, excluding patients with chronic kidney disease, re-LT, or mortality in the first 10 days.	Mortality.	RBC transfusion requirement; Surgical time.	–
Nacif et al., 2018 [24]	Retrospective, single-center	Any indication for LT.	Surgical time; Warm ischemia time; Serum creatinine level at 24 and 72 h; ICU and hospital stay; Incidence of Clavien–Dindo complication score ≥ 3; Overall survival.	RBC, platelet, and FFP transfusions; Cold ischemia time.	Total ischemia time.
Yl et al., 2024 [32]	Randomized, single-center	Live-donor LT, excluding patients with ALF or ACLF, portal vein thrombosis, portal cavernomas, large portosystemic shunts, TIPS, and Budd–Chiari syndrome.	SBP, DBP, and mean arterial pressure before reperfusion; Intraoperative vasopressor requirements; Intraoperative urinary output; Risk of IVC injury; Intraoperative blood loss; Surgical time; Absolute need and amount of blood product transfusions; Portal pressure gradient after implantation; Time for lactate levels normalization; Enteral feed toleration.	Intraoperative and 1st POD lactate levels; Cold and warm ischemia time; Major morbidity; 90-day mortality; AKI incidence; Ascites drainage, ICU and hospital stay.	Anhepatic phase duration.
Pietersen et al., 2025 [23]	Retrospective, single-center	Any indication for LT, excluding ALF, patients receiving a split liver, or with prior kidney injury.	Blood product requirements.	FFP transfusion requirements; Volume of cell saver return; Serum creatinine levels up to 7th POD; Development of AKI; Need for renal replacement therapy; Incidence of Clavien–Dindo complication score ≥ 3a; 1-year graft and patient survival.	Warm ischemia time.

Annotations: “Positive” means that PCS has a positive, statistically significant impact on the mentioned outcome, i.e., reduced need for RBC transfusions, improved urinary output, and less reduced cardiac output. “Neutral” means there is no significant difference between the PCS and no-PCS group. “Negative” means that the PCS group was negatively impacted compared to the no-PCS group. “Absolute need for transfusions” means any amount of transfusions vs. no transfusions at all; “amount” refers to the quantity of blood transfused. * Only patients receiving a cavo-cavostomy using the suprahepatic graft caval vein, end-to-side, were included. Abbreviations: ACLF—acute on chronic liver failure; AKI—acute kidney injury; ALF—acute liver failure; DBP—diastolic blood pressure; D-MELD—donor MELD; DRI—donor risk index; ECD—extended donor criteria; FFP—fresh frozen plasma; ICU—intensive care unit; IVC—inferior vena cava; LT—liver transplantation; MELD—model for end-stage liver disease; PCS—portocaval shunt; POD—postoperative day; PV—portal vein; RBC—red blood cell; SBP—systolic blood pressure; TIPS—transjugular intrahepatic portosystemic shunt.

**Table 3:** Summary of the main and more common indications for PCS.

Main Indications for PCS
Complex pedicle dissection Severe portal hypertension Elevated intraoperative bleeding risk Fulminant hepatitis Lack of spontaneous portosystemic shunts

## Data Availability

No new data were created or analyzed in this study.

## Abbreviations

The following abbreviations are used in this manuscript:

EAD	Early Allograft Disfunction
ECD	Extended-Criteria Donor
IMV	Inferior Mesenteric Vein
IRI	Ischemia–Reperfusion Injury
IVC	Inferior Vena Cava
LT	Liver Transplantation
PCS	Portocaval Shunt
PRS	Postreperfusion Syndrome
PV	Portal Vein
VVB	Veno-venous Bypass
